# Application of Quantitative Structure-Activity Relationships in the Prediction of New Compounds with Anti-Leukemic Activity

**DOI:** 10.3390/ijms241512258

**Published:** 2023-07-31

**Authors:** Cristian Sandoval, Francisco Torrens, Karina Godoy, Camila Reyes, Jorge Farías

**Affiliations:** 1 Escuela de Tecnología Médica, Facultad de Salud, Universidad Santo Tomás, Los Carreras 753, Osorno 5310431, Chile; 2 Departamento de Ingeniería Química, Facultad de Ingeniería y Ciencias, Universidad de La Frontera, Temuco 4811230, Chile; 3 Departamento de Ciencias Preclínicas, Facultad de Medicina, Universidad de La Frontera, Temuco 4811230, Chile; 4 Institut Universitari de Ciència Molecular, Universitat de València, 46071 València, Spain; torrens@uv.es; 5 Nucleo Científico y Tecnológico en Biorecursos (BIOREN), Universidad de La Frontera, Temuco 4811230, Chile; karina.godoy@ufrontera.cl; 6 Carrera de Tecnología Médica, Facultad de Medicina, Universidad de La Frontera, Temuco 4811230, Chile; c.reyes24@ufromail.cl

**Keywords:** bone marrow microenvironment, cancer, leukemia, myeloproliferative disorders, model

## Abstract

Leukemia invades the bone marrow progressively and, through unknown mechanisms, outcompetes healthy hematopoiesis. Protein arginine methyltransferases 1 (PRMT1) are found in prokaryotes and eukaryotes cells. They are necessary for a number of biological processes and have been linked to several human diseases, including cancer. Small compounds that target PRMT1 have a significant impact on both functional research and clinical disease treatment. In fact, numerous PRMT1 inhibitors targeting the S-adenosyl-L-methionine binding region have been studied. Through topographical descriptors, quantitative structure-activity relationships (QSAR) were developed in order to identify the most effective PRMT1 inhibitors among 17 compounds. The model built using linear discriminant analysis allows us to accurately classify over 90% of the investigated active substances. Antileukemic activity is predicted using a multilinear regression analysis, and it can account for more than 56% of the variation. Both analyses are validated using an internal “leave some out” test. The developed model could be utilized in future preclinical experiments with novel drugs.

## 1. Introduction

Only 40% of <60 years old and 10% of >60 years old individuals have achieved long-term survival from acute myeloid leukemia (AML) [1]. The clones of leukemia that drive the disease’s persistence and recurrence are resistant to current chemotherapy and targeted treatments. Most AML patients have elevated expression of FLT3, a tyrosine kinase that may play a role in the etiology of the disease [2]. The continual ligand-independent activation of FLT3 kinase that this mutation causes makes it a promising therapeutic target [3]. When administered alone, tyrosine kinase inhibitors (TKIs) have extremely short-lived therapeutic effects and are only able to partially suppress AML cell proliferation [3,4]. Improved clinical response for FLT3-ITD+ AML patients requires the rapid development of effective combination treatments, such as TKI therapy.

To regulate signal transduction and protein–protein interactions, arginine residues can be modified into asymmetric dimethylarginine (ADMA) by adding two methyl groups to a single guanidino nitrogen [5,6]. About 85% of arginine methylation activities in human cells are carried out by PRMT1, which deposits an ADMA mark onto substrates [7]. PRMT1 methylates not just histones but also proteins, including RUNX1 and EGFR, and has been linked to processes as varied as cell proliferation, survival, and differentiation [5,8,9]. PRMT1 methylates AML1-ETO9, increasing its transcriptional activity in murine leukemia transformed by this fusion oncoprotein [9]. Recent research indicates that the oncogenic fusion protein MLL-GAS7 or MLL-EEN recruits PRMT1 to methylate H4R3, hence sustaining leukemic transcriptional pathways [10].

The PRMTs facilitate the transfer of methyl groups from the S-adenosylmethionine molecule to the guanidino nitrogen atoms of arginine. Based on how the arginine residues are methylated, methylarginines can be divided into three distinct forms: ω-NG,NG-asymmetric dimethylarginine (aDMA), ω-NG,N′G-symmetric dimethylarginine (sDMA), and ω-NG-monomethylarginine (MMA). All PRMTs catalyze MMA, which is then used by Type I PRMTs (PRMT1, PRMT2, PRMT3, PRMT4, PRMT6, and PRMT8) to catalyze the production of aDMA or by Type II PRMTs (PRMT5 and PRMT9) to catalyze the formation of sDMA [5,6]. The PRMT7 is the only member of the Type III enzyme subclass that catalyzes MMA formation [5,6,7].

The PRMTs have been implicated in human tumorigenesis in numerous studies. PRMT enzymatic activity is necessary for many cellular processes in hematological malignancies, such as the activation of cell cycle and proliferation, inhibition of apoptosis, DNA repair processes, RNA splicing, and transcription by methylating histone tails’ arginine [11,12,13]. In human malignancies, elevated PRMT expression is associated with aggressive clinical features and a poor prognosis [10,14]. PRMT1 overexpression promotes the survival and invasion of cancer cells, whereas PRMT1 suppression inhibits the proliferation of cancer cells [8,15,16,17,18]. In addition, growing evidence suggests that PRMT1 plays key roles in malignant hematopoiesis [19].

Nowadays, various in silico technologies are utilized to design and assess the efficacy of new medications, one of which is molecular topology, specifically molecular connectivity [20], which has proven to be useful in quantitative structure-activity relationship (QSAR) models. One of the most exciting features of molecular topology is the ease with which topological descriptors can be calculated. Each structure is described as a hydrogen-depleted network, with vertices representing atoms and edges representing bonds. Manipulation of such matrices gives multiple sets of integers known as topological indices [20], which have been shown to be capable of a simple and effective characterization of molecular structure. When these indices are chosen correctly, it is possible to obtain a highly specific characterization of each chemical compound, which can then be used in QSAR models [21,22,23,24,25].

In this way, the topological indices have shown their value for the selection and creation of novel pharmaceuticals [26,27], especially as antimalarial [28], antiviral [29], antihistaminic [30], hypoglycemic [31], analgesics [32,33], antituberculosis [34] and antileukemic drugs [35]. However, there are numerous limitations to consider when using QSAR modeling in the pharmaceutical business [36,37]. Due to the large number of variables involved in QSAR data—hundreds of thousands of chemicals, each represented by a unique set of descriptors, fingerprints that are typically very sparse, and some features that are highly correlated—it is anticipated that the dataset contains some mistakes since associations are examined by in situ studies.

As a result of these limitations, QSAR-based model prediction has been controversial. This has led to QSAR prediction being used along with machine-learning algorithms. For QSAR prediction, previous studies have turned to linear regression models [38], leave-one-out cross-validation [39], and multilinear regression analysis [40]. Our research examined the activities of a set of PRMT1 inhibitors of leukemia cell proliferation to develop QSAR models of prediction using molecular topology, linear discriminant analysis, and multilinear regression analysis. A screening method was also developed to identify novel compounds with the potential for greater bioactivity.

## 2. Results

### 2.1. Linear Discriminant Analysis

The search for an applicable mathematical-topological model to predict antileukemic activity was conducted in two phases. First, a discriminant function was chosen to differentiate between active and inactive antileukemic compounds. Second, the acquisition of a topological function capable of quantifying the efficacy of the activity in terms of IC_50_. Both functions would constitute the framework of the mathematical model that permits the search for and selection of new potent antileukemic compounds.

To obtain the discriminant function, a linear discriminant analysis was applied to 17 compounds. The set consisted of both active and inactive compounds. The IC_50_ ≤ 1 M/10^5^ concentration denotes the active compounds, whereas the IC_50_ > 1 M/10^5^ concentration denotes the inactive compounds. The selected discriminant function was:

(1) $$\mathrm{Discriminant}\;\mathrm{function}=-14.123+\left(0.848\times \mathrm{nDB}\right)+\left(1.680\times \mathrm{nN}\right)\;–\left(38.327\times \mathrm{GGI}9\right)+\left(392.190\times \mathrm{JGI}4\right),\;\;N=17,\;\lambda \;(\mathrm{Wilk}’s\;\mathrm{Lambda})=0.172,\;F\;(4,15)=14.442,\;p\;<\;0.001$$

In Equation (1), there were topological descriptors that evaluated the constitutional characteristics of each compound (nDB), atom count (nN), and topological charge (GGI9 and JCI4).

**Figure 1:**
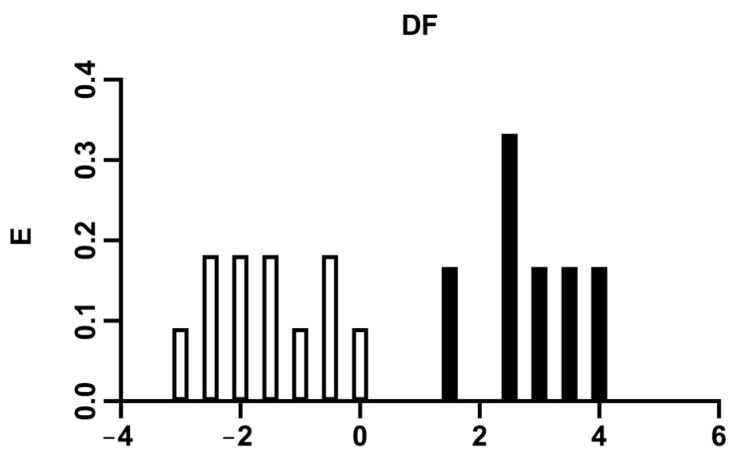
Pharmacological distribution diagram for antileukemic activity by plotting expectancy (*E*) vs. discriminant function (DF) (Equation (1)). The black bars represent the compounds with IC_50_ ≤ 1 M/10^5^, and the white bars, the compounds with IC_50_ > 1 M/10^5^.

Figure 1 depicts the antileukemic activity distribution diagram using the discriminant function (white bars represent inactive sets, and black bars represent active sets).

### 2.2. Multilinear Regression Analysis

Multiple linear regression allows one to generate a linear model in which the value of the dependent or response variable, pIC_50_ (pIC_50_ = −log[IC_50_]), is predicted from a set of independent variables called topological indices. To perform the multilinear regression analysis, compounds with quantitative IC_50_ values in M/10^5^ units were used. In addition, the **17a** compound was removed as it is an outlier. The number of compounds needed to carry out the analysis was reduced to 16. The selected function was:

(2) $${\mathrm{pIC}}_{50}\mathrm{predicted}=-42.089+\left(314.536\times \mathrm{JGI}4\right)-\left(25.006\times \mathrm{GGI}7\right)+\left(59.732\times \mathrm{Mv}\right)+\left(0.450\times X0v\right),\;\;N=16,\;R^{2}=0.898,\;Q^{2}=0.863,\;\mathrm{SEE}=0.646,\;p=0.009$$

**Figure 2:**
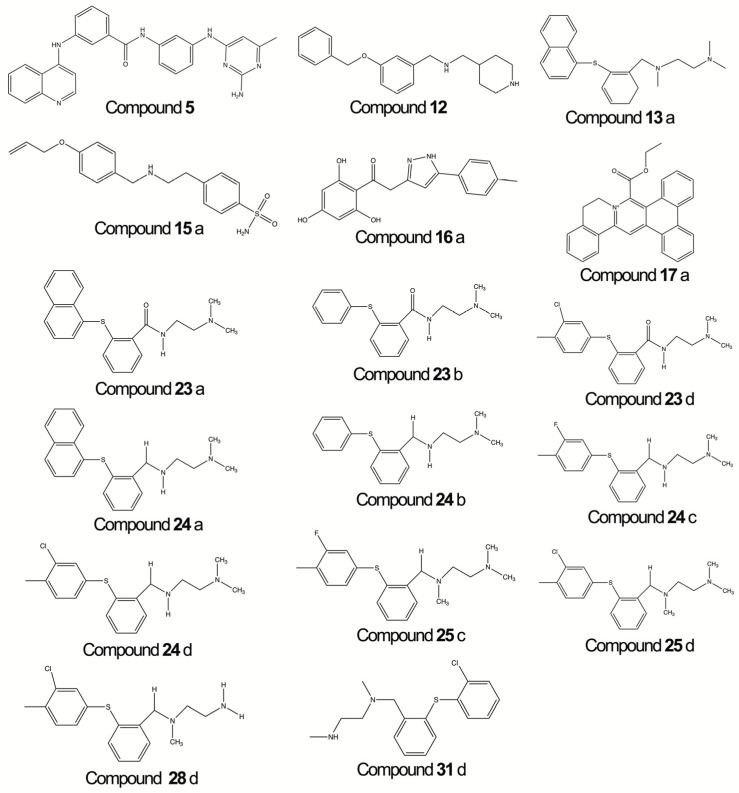
Chemical structures of the studied compounds.

The predictive equation, Equation (2), exhibits an R^2^ value above 0.80 (0.898), which explains over 89% of the variance. The descriptors selected are the mean topological charge index (JGI4), topological charge index (GGI7), mean atomic van der Waals volume (Mv), and the valence connectivity index (X0v). Particularly, the JGI4 index measures the charge transfer between pairs of atoms and, consequently, the global charge transfer in the molecule (e.g., the dipole moment) [32,33]; GGI7 index features the charge transfer between a pair of atoms, accounting for the overall charge transfer within the molecule [26], Mv index shows the sum of the van der Waals volumes by the number of atoms, and the X0v index, the connectivity (Figure 2).

**Figure 3:**
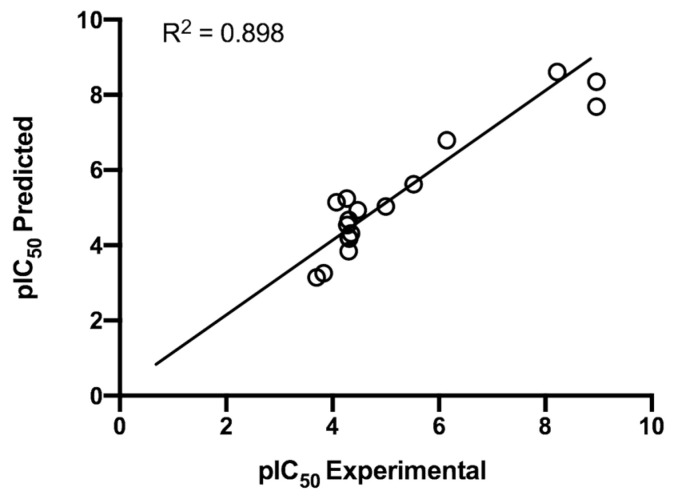
Prediction of growth inhibition of leukemia cell proliferation (M/10^5^) for the PRMT1 derivatives. Graphic representation of experimental log IC_50_ against log IC_50_ calculated from Equation (2).

Table 1 and Figure 3 summarize the predictions made for each compound in the training set using Equation (2). Overall, we can conclude that there is an acceptable level of efficacy because 56.3% of compounds exhibit residuals shorter than ±1SEE. The value of the greatest residual compound, compound **25d**, is −1.271. Linear discriminant analysis categorizes this compound as active, which implies that either the experimental IC_50_ is correct or the topological model used here is a valid classification for the compound’s antileukemic activity.

**Figure 4:**
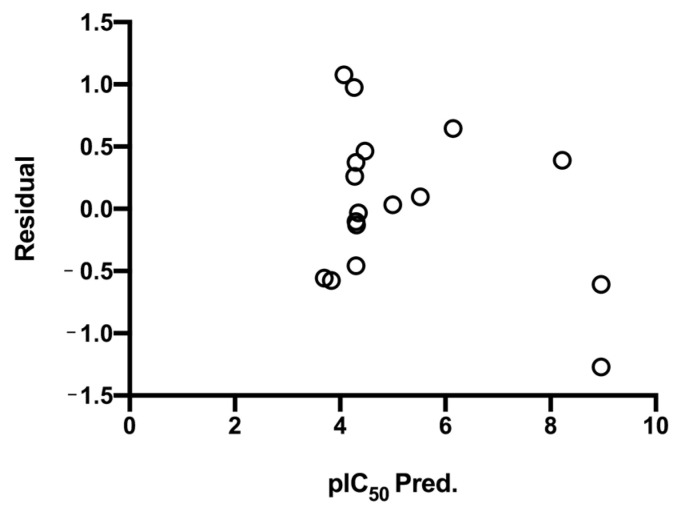
Graphic representation of the residual of pIC_50_ against predicted pIC_50_ obtained from the selected prediction function, Equation (2). The interval in which they are presented corresponds to ±2SD.

Equation (2) was validated using leave-one-out cross-validation and an external test (Figure 4). The coefficient of prediction, Q^2^ = 0.863, shows that Equation (2) has a high robustness. The test results are displayed in Table 1, where some anticipated logIC_50_ values are greater than 1.0, suggesting that the estimated IC50 value is less than 1 M/10^5^.

## 3. Discussion

One of the most common somatic mutations in AML is FLT3-ITD, which is seen in 25% of AML patients and is linked with a poor prognosis [3]. TKI therapy has shown relatively limited efficacy for individuals with AML, and recurrence is linked to the survival of FLT3-ITD+ AML clones [3,44]. Therefore, novel therapies are required to eradicate FLT3-ITD+ AML cells. In fact, PRMT1 specifically binds oncogenic FLT3, catalyzes its protein methylation, and hence promotes the survival and proliferation of FLT3-ITD+ AML cells [42]. In particular, FLT3 methylation levels are maintained in AML cells even after TKI treatment, and limiting this activity with a pharmacological inhibitor improved FLT3-ITD+ AML cell elimination by a TKI, suggesting that PRMT1 inhibition might function as a therapeutic for AML patients with FLT3-ITD [45].

The expression of PRMT1 is consistently high in malignant tumors [10,18,46,47,48]. However, the role it plays in these situations is likely to be determined by the role(s) of its substrate(s) [10,18,46,47,48]. Loss of PRMT1 activity inhibits MLL-GAS7- or MLL-EEN-driven leukemogenesis, according to recent research [10]. Specifically, PRMT1 methylates H4R3, which is critical for oncogenic transcriptional programs. There is growing evidence to show that, in contrast to the FLT3 WT receptor, the FLT3-ITD protein preferentially localizes intracellularly [49,50,51]. As a mostly nuclear and cytoplasmic protein, PRMT1 preferentially interacts with FLT3-ITD over FLT3 WT protein [52]. As a result, PRMT1 has significantly better access to FLT3-ITD protein than to FLT3 WT protein.

About 30% of individuals with AML have FLT3 changes, such as internal tandem duplication and point mutations within the tyrosine kinase domain (TKD) [53,54]. Inhibitors of FLT3 kinase-like sorafenib, quizartinib, and gilteritinib have been utilized in clinical practice [55]. However, clinical responses to these drugs are transient because of high rates of relapse and drug resistance after treatment, which contributes to disease progression and poor overall survival [4,56]. Therefore, finding effective compounds to overcome drug resistance is an urgent problem.

The steps involved in developing a QSAR model are as follows: (I) choosing a set of molecules that cover a wide range of chemical space and have verified bioactivity; (II) making 2D/3D structures of the molecules and optimizing them with the right molecular mechanics; (III) calculating molecular descriptors and pruning data with a good statistical method; and (IV) making a QSAR model with the right method. We used quantitative structure-activity relationships to computationally screen 17 PRMT1 inhibitors of leukemia cell proliferation.

According to Mitteroecker and Bookstein [57], linear discriminant analysis is a statistical method for classifying data into two or more groups using a linear formalism [38]. Linear discriminant analysis, like multiple linear regression, creates a predicted association between categorical and continuous descriptor values. Another technique that looks for differentiation between groups is linear discriminant analysis. The equation below defines an arbitrary discriminant function: DF = C0 + C1X1 + C2X2 + … + CKXK where, X1, X2…Xk represents the predictor scores of the total k variables and C1, C2…Ck represents their respective weights. Thus, if the discriminant function was greater than zero, a given compound was selected as a potential antileukemic agent; otherwise, it was categorized as “inactive.” The classification matrix was highly significant for the set (99.9% correct prediction for the active group, five out of five correctly classified, and 91.7% for the inactive group, 11 out of 12 correctly classified; Table 1).

It is evident that the regions with the least overlap for compounds with theoretical antileukemic activity occur when discriminant function >1, indicating that the highest activity expectation occurs over this value. If discriminant function >0 and <1, a compound will be classified as non-classified (NC). Based on the outcomes of linear discriminant analysis and multilinear regression (Equations (1) and (2)), a topological model for the search for novel antileukemic agents can be formulated. The search was made for PRMT1 inhibitor compounds according to the following requirements. If the discriminant function is >1 and <5, and pIC50 is greater than 9, then the compound is labeled as potentially antileukemic. Otherwise, the compound would be considered inactive.

Antileukemic activity assays must support these suggestive findings in order to enable the validation or evaluation of the suggested model and to act as a useful tool in the search for novel compounds with higher activity against leukemia cell proliferation.

The QSAR method’s main strength is that it can anticipate the characteristics of novel chemical compounds without first having to synthesize and test them. The chemical, industrial, medicinal, biological, and environmental fields all make use of this method for predicting physicochemical qualities [58]. In addition, QSAR techniques reduce costs and speed up the time it takes to create novel compounds for application as medications, materials, and additives, among others [59]. In contrast, molecular docking is a computer tool for assessing the affinity of active site residues for a given molecule or molecules [60]. The drug development industry makes use of molecular docking as a time-saving approach to examine ligand-target binding compatibility [61]. So, in future studies, molecular binding could help us to examine the ligand-target binding compatibility.

## 4. Materials and Methods

### 4.1. Analyzed Compounds and Tests Carried Out

In this QSAR study, we used the work of Wang et al. [41] to choose a group of 17 PRMT1 inhibitors of leukemia cell proliferation. As a reference, we used compound **5**, which has a half-maximal inhibitory concentration (IC_50_) against leukemia cell proliferation of 1 M/10^5^. All these compounds have demonstrated inhibitory activity against leukemia cell proliferation, which has been previously tested and experimentally verified [41,42,43]. The respective IC_50_ (M/10^5^) concentration values and their chemical structures are available (Table 1).

It is important to note that each molecule still has the same number as it did in the original work from which the compounds were taken [41,42,43]. To draw the chemical structure of the molecules, the ChemDraw^®^ Professional 22.0.0.22 software was used (Figure 2).

### 4.2. Molecular Descriptors

Well-known topological descriptors have been used in this work: Subgraph Randić–Kier–Hall-like indices up to the fourth order (^m^χ_t_, ^m^χ_t_^v^) [62,63], topological charge indices, up to the fifth order (J_m_, G_m_, J^v^_m_, G^v^_m_) [31,32], quotients and differences between valence and non-valence connectivity indices (^m^C_t_ = ^m^χ_t_/^m^χ^v^_t_ and ^m^D_t_ = ^m^χ_t_ − ^m^χ^v^_t_) [64,65]. Each compound was characterized by a set of 100 descriptors. Table 2 shows the symbol, name, and definition of each descriptor. All descriptors used in this work were obtained with Alvascience^®^ software, version 2 [66]. The descriptors are available on https://doi.org/10.6084/m9.figshare.22784939.v1 (accessed on 23 June 2023)

### 4.3. QSAR Algorithms

#### Linear Discriminant Analysis

Linear discriminant analysis is an algorithm that allows us to distinguish between two or more categories or objects by means of a linear function. In our case, it is about differentiating or discriminating between active and inactive compounds according to the values of the descriptors of their molecules [25].

Two sets of compounds: The first with proven activity against leukemia cell proliferation (in our case, all the compounds with IC_50_ ≤ 1 M/10^5^), and the second comprised inactive compounds (IC_50_ > 1 M/10^5^) were considered for the analysis. The percentage of correct classifications tested the discriminant ability in each group. Linear discriminant analysis was performed using the SPSS^®^ software, version 20 (IBM Corp., Armonk, NY, USA). The selection of the descriptors was based on the Fisher-Snedecor parameter, and the classification criteria were based on the shortest Mahalanobis distance (distance to the corresponding centroid) [67]. The statistical program selects the variables used for the calculation of the discriminant function in stepwise way. In fact, it reviews all the variables, and the one that contributes the most to the discrimination of the groups will be included in the model, while the variable that contributes the least to the prediction will be eliminated. The quality of the discriminant function was evaluated by Wilk’s lambda parameter, λ, which is a statistic of multivariate analysis of variance that tests the equality of group means for the variable(s) in the discriminant function [68].

From the selected discriminant function, the pharmacological activity distribution diagram was drawn. This diagram was pictured just to establish the intervals of the discriminant function in which the expectancy, *E*, of finding antileukemic compounds is maximum. Pharmacological activity distribution diagrams are histogram-like plots of connectivity functions in which expectancies appear on the ordinate axis. For each arbitrary interval of any function, the expectancy of activity *Ea* is defined as *Ea* = *a*/(*I* + 1), where “*a*” is the quotient between the number of active compounds in the interval divided by the total number of active compounds, and “i” is the number of inactive compounds in the interval divided by the total number of inactive compounds. The expectancy of inactivity, *Ei*, is defined in a symmetrical way as *Ei* = *i*/(*a* + 1). This representation allows us to see the areas in which the overlap is minimal, as well as to determine the intervals of the discriminant function, where the probability of finding new active compounds is maximum in relation to the choice of a false active [69].

### 4.4. Multilinear Regression Analysis

The IC_50_ values have been softened as pIC_50_ = −logIC_50,_ as is usual in QSAR studies. The regression equation was obtained by correlating the experimental pIC_50_ values with the topological index by multilinear regression analysis through the SPSS^®^ software (IBM Corp., Armonk, NY, USA). The judgment for the selection of variables consisted of using the group with the least number of variables to avoid overfitting, and the value of the multiple correlation coefficient, R^2^, was high (R^2^ > 0.8), and the standard error of the estimate was minimal. To accept the prediction function, an internal validation test was performed. The validation of the prediction function is performed through an internal cross-validation of the leave-one-out type; that is, determine the prediction coefficient (Q^2^).

#### Leave-One-Out Cross-Validation

Each compound is removed from the model, and the activity value, pIC_50_, is recalculated with the other compounds and descriptors from the selected equation. The process is repeated as many times as compounds are studied [70]. With the predicted values, the value of the prediction coefficient (Q^2^) is determined and compared with the value of R^2^. The values of Q^2^ > 0.7 show us that and, therefore, that the function obtained is robust and the selected model is of good quality.

### 4.5. Limitations

The purpose of this review was (1) to examine the activities of a set of PRMT1 inhibitors to develop QSAR models of prediction using molecular topology, linear discriminant analysis, and multilinear regression analysis and (2) to develop a screening method to identify novel compounds with greater antileukemic bioactivity. However, our in silico study should be replicated using in vitro and in vivo models with PRMT1 inhibitors must be made. In addition, future studies should perform molecular dynamics studies for the top three compounds and analyze the thermodynamic parameters, such as the free energy of binding using, for example, molecular mechanics/Poisson–Boltzmann surface area. Finally, molecular docking tools for this study of protein-ligand interactions and virtual screening should be performed.

## 5. Conclusions

Successfully using molecular topology to identify a QSAR model to predict the antileukemic activity of a group of PRMT1 inhibitor compounds. All employed molecular descriptors are graph-theoretic in nature. The mathematical model utilized in this study retains the primary structural features involving the correlated property, IC50, and is therefore applicable to the virtual screening of databases for the discovery of new active compounds. In the next stage of research, multiple PRMT1 inhibitor compounds should be compiled into a virtual library for computationally pursuing and optimizing antileukemic activity against leukemia cell proliferation. Significant enhancements to the activity have been achieved.

## Figures and Tables

**Table 1:** Classification results were obtained from linear discriminant analysis and multilinear regression for each compound analyzed.

Comp.	SMILES	IC_50_ Exp. ^a^ (M/10^5^)	pIC_50_ Exp.	Class (Exp.)	Prob. Activ. ^b^	DF ^c^	Class (Pred.)	pIC_50_ Pred. ^d^
**5**	NC1=NC(NC2=CC=CC(NC(C3=CC=CC(NC4=CC=NC5=CC=CC=C54)=C3)=O)=C2)=CC(C)=N1	1	5.000	A	1.000	3.743	A	5.033
**12**	C1(OCC2=CC=CC=C2)=CC=CC(CNCC3CCNCC3)=C1	14.7	3.833	I	<0.001	−2.443	I	3.258
**13a**	CN(CCN(C)C)CC1=C(C=CCC1)SC2=C3C(C=CC=C3)=CC=C2	0.3	5.523	A	0.971	1.586	A	5.620
**15a**	O=S(N)(C1=CC=C(CCNCC(C=C2)=CC=C2OCC=C)C=C1)=O	8.5	4.071	I	<0.001	−0.922	I	5.147
**16a**	OC1=CC(O)=C(C(CC2=NNC(C3=CC=C(C)C=C3)=C2)=O)C(O)=C1	4.9	4.310	I	<0.001	−2.225	I	4.180
**17a**	O=C(OCC)C1=C(C2=CC=CC=C2C3=CC=CC=C43)C4=CC5=[N+]1CCC6=C5C=CC=C6	5.3	4.276	I	0.038	0.024	NC	4.536
**23a**	O=C(N([H])CCN(C)C)C1=C(C=CC=C1)SC2=CC=CC3=C2C=CC=C3	>20	<3.699	I	<0.001	−3.163	I	3.142
**23b**	O=C(N([H])CCN(C)C)C1=C(C=CC=C1)SC2=CC=CC=C2	>5	<4.301	I	<0.001	−2.123	I	4.199
**23d**	O=C(N([H])CCN(C)C)C1=C(C=CC=C1)SC2=CC=C(C)C(Cl)=C2	>5	<4.301	I	<0.001	−1.255	I	3.843
**24a**	[H]C(N([H])CCN(C)C)C1=C(C=CC=C1)SC2=CC=CC3=C2C=CC=C3	3.4	4.469	I	<0.001	−2.431	I	4.932
**24b**	[H]C(N([H])CCN(C)C)C1=C(C=CC=C1)SC2=CC=CC=C2	>5	<4.301	I	<0.001	−1.527	I	4.673
**24c**	[H]C(N([H])CCN(C)C)C1=C(C=CC=C1)SC2=CC=C(C)C(F)=C2	4.5	4.347	I	0.008	−0.338	I	4.314
**24d**	[H]C(N([H])CCN(C)C)C1=C(C=CC=C1)SC2=CC=C(C)C(Cl)=C2	5.4	4.268	I	0.008	−0.338	I	5.244
**25c**	[H]C(N(C)CCN(C)C)C1=C(C=CC=C1)SC2=CC=C(C)C(F)=C2	0.071	6.149	A	0.999	2.299	A	6.795
**25d**	[H]C(N(C)CCN(C)C)C1=C(C=CC=C1)SC2=CC=C(C)C(Cl)=C2	0.039	8.959	A	0.999	2.299	A	7.688
**28d**	[H]C(N(C)CCN([H])[H])C1=C(C=CC=C1)SC2=CC=C(C)C(Cl)=C2	0.00011	8.959	A	0.999	2.886	A	8.352
**31d**	CNCCN(CC1=CC=CC=C1SC2=CC=CC=C2Cl)C	0.0006	8.222	A	1.000	3.931	A	8.612

^a^ Experimental IC_50_ values taken from the work of Wang et al. [41], Valente et al. [42], Xie et al. [43]; ^b^ probability that the compound is active; ^c^ DF values obtained with Equation (1); ^d^ Activity values predicted with Equation (2). A: active; I: inactive; DF: discriminant function; NC: non-classified. The compound classified as an outlier has not been predicted.

**Table 2:** Symbol, name, and definition of each descriptor used in this study.

Symbol	Name	Definition	
*^k^*χ_t_, *k* = 0–4 and t = p, c, pc	Randic-like indices of order *k* and type path (p), cluster (c), and path-cluster (pc)	$k_{\chi t}=\overset{k_{nt}}{\underset{j=1}{\sum }}{\left(\underset{i\;\in \;S_{j}}{\prod }\delta_{i}\right)}^{-1/2}$	$\mathrm{where}\;\delta_{i}$$\;\mathrm{is}\;\mathrm{the}\;\mathrm{number}\;\mathrm{of}\;\mathrm{bonds},\;\sigma \;\mathrm{or}\;\pi ,\;\mathrm{of}\;\mathrm{the}\;\mathrm{atom}\;i\;\mathrm{to}\;\mathrm{non}\text{-}\mathrm{hydrogen}\;\mathrm{atoms}.\;S_{j}$ is the *j*th sub-structure of order *k* and type t
$k_{\chi_{t}^{v}}$, *k* = 0–4 and t = p, c, pc	Kier–Hall indices of order *k* and type path (p), cluster (c), and path-cluster (pc)	$k_{\chi_{t}^{v}}=\overset{k_{nt}}{\underset{j=1}{\sum }}{\left(\underset{i\;\in \;S_{j}}{\prod }\delta_{i}^{V}\right)}^{-1/2}$	$\mathrm{where}\;\delta_{i}^{V}$$\;\mathrm{is}\;\mathrm{the}\;\mathrm{Kier}\text{-}\mathrm{Hall}\;\mathrm{valence}\;\mathrm{of}\;\mathrm{the}\;\mathrm{atom}\;i.\;S_{j}$ is the *j*th sub-structure of order *k* and type t
G*_k_*, *k* = 1–5	Topological charge indices of order *k*	$G_{k}=\overset{N-1\;}{\underset{i=1}{\sum }}\overset{N}{\underset{j=i+1}{\sum }}\left|M_{ij}-\left.M_{ji}\right|\to \delta \;\left(k,\;D_{ij}\right)\right.$	where M = AQ is the product of the adjacency and inverse square distance matrices for the hydrogen-depleted molecular graph. D is the distance matrix. *δ* is the Kronecker delta
$G_{k}^{V}$, *k* = 1–5	Valence topological charge indices of order *k*	$G_{k}^{V}=\overset{N-1\;}{\underset{i=1}{\sum }}\overset{N}{\underset{j=i+1}{\sum }}\left|M_{ij}^{V}-\left.M_{ji}^{V}\right|\to \delta \;\left(k,\;D_{ij}\right)\right.$	where M^V^ = A^V^Q is the product of the electronegativity-modified adjacency and inverse square distance matrices for the hydrogen-depleted molecular graph. D is the distance matrix. *δ* is the Kronecker delta
$J_{k}$, *k* = 1–5	Normalized topological charge indices of order *k*	$J_{k}=\frac{G_{k}}{N-1}$	
J^v^, *k* = 1–5	Normalized valence topological charge indices of order *k*	$J_{k}^{V}=\frac{G_{k}^{V}}{N-1}$	
*^k^D*t, *k* = 0–4 and t = p, c, pc	Connectivity differences of order *k* and type path (p), cluster (c), and path-cluster (pc)	${}^{k}D_{t}={}^{k}\chi_{t}-{}^{k}\chi_{t}^{V}$	
^*k*^C_*t*_, *k* = 0–4 and t = p, c, pc	Connectivity quotients of order *k* and type path (p), cluster (c), and path-cluster (pc)	${}^{k}C_{t}=\frac{{}^{k}\chi_{t}}{{}^{k}\chi_{t}^{V}}$	

## Data Availability

The data presented in this study are available at “https://doi.org/10.6084/m9.figshare.22784939.v1” (accessed on 23 June 2023).
